# Meaninglessness and monotony in pandemic boredom

**DOI:** 10.1007/s11097-023-09888-0

**Published:** 2023-01-20

**Authors:** Emily Hughes

**Affiliations:** Department of Philosophy, University of York, Heslington, York, YO10 5DD UK

**Keywords:** Boredom, Covid-19, Pandemic, Heidegger, Meaninglessness, Time

## Abstract

Boredom is an affective experience that can involve pervasive feelings of meaninglessness, emptiness, restlessness, frustration, weariness and indifference, as well as the slowing down of time. An increasing focus of research in many disciplines, interest in boredom has been intensified by the recent Covid-19 pandemic, where social distancing measures have induced both a widespread loss of meaning and a significant disturbance of temporal experience. This article explores the philosophical significance of this aversive experience of ‘pandemic boredom.’ Using Heidegger’s work as a unique vantage point, this article draws on survey data collected by researchers in an ongoing project titled ‘Experiences of Social Distancing During the Covid-19 Pandemic’ to give an original phenomenological interpretation of the meaninglessness and monotony of pandemic boredom. On a Heideggerian interpretation, pandemic boredom involves either a situative confrontation with relative meaninglessness that upholds our absorption in the everyday world, or an existential confrontation with absolute meaninglessness that forces us to take up the question of our existence. Arguing that boredom during the pandemic makes this distinction difficult to sustain, I consider some of the ways in which pandemic boredom might be seen to expose and then exceed the distinctive methodological limitations of Heidegger’s philosophical interpretation of boredom.

## Introduction

Boredom is an affective experience that can involve pervasive feelings of meaninglessness, emptiness, restlessness, frustration, weariness and indifference, as well as the slowing down of time.^1^ A modern iteration of the pre-modern moral concept of *acedia*, boredom has proliferated in the secularized and technologized societies of modernity and ‘boredom studies’ has gained momentum in many disciplines, including philosophy, as a result.^2^ This preoccupation has been intensified by the recent Covid-19 pandemic, where social distancing measures have induced both a widespread loss of meaning,^3^ and a significant disturbance of temporal experience.^4^ Constituted by pervasive experiences of meaninglessness and monotony, boredom is unquestionably one of the paradigmatic emotions of this “half-assed end of the world.” As Mark O’Connell writes for *The Guardian*:

There has been no grand systemic collapse, but there has been a collapse of the experience of time, and of the sense of its meaning. The flatness of the days, the endless sameness, is building towards some cumulative emotional effect, and we have not yet begun to take the measure of it. I am increasingly catching myself in the act of wishing away months of my life, of wanting the time between now and whenever this stasis ends to pass as quickly as possible (O’Connell, 2021).

Forced to endure this “boring apocalypse” (Grant, 2021), the philosophical significance of this languishing presents itself with a renewed urgency. Part of what has made ‘pandemic boredom’^5^ particularly distressing, is that we have not known how much time there will be between ‘now’ and ‘whenever this stasis ends’ and, indeed, whether or not it will in fact end. At a time when we are being forced to confront our collective contingency and lack of control, pandemic boredom prompts us to consider the significance of the aversive experience of boredom in a new light.^6^

My first aim in this article is to give an original phenomenological interpretation of the meaninglessness and monotony of pandemic boredom in order to understand its philosophical significance. Whilst contributing to extant work on the pandemic which shows how phenomenology can shed light on pandemic experience, I also consider how pandemic experience itself can lead to a re-evaluation of phenomenological frameworks. Using Martin Heidegger’s influential account of boredom as a unique vantage point, I will consider whether pandemic boredom involves a situative confrontation with relative meaninglessness that merely upholds our absorption in the everyday world; or whether it might also allow for an existential confrontation with absolute meaninglessness that forces us to take up the question of our existence.^7^ That is, can pandemic boredom be seen to wrench us out of our familiar world, force us to recognise our finitude, and take up responsibility for shaping our lives in a meaningful way? Proposing that boredom during the pandemic makes this distinction difficult to sustain, the second and more critical aim of this article is to consider some of the ways in which pandemic boredom might be seen to expose and then exceed the distinctive methodological limitations of Heidegger’s philosophical interpretation of boredom.

To set up this analysis I firstly give a philosophical account of boredom. Drawing primarily on Martin Heidegger’s paradigmatic study of boredom in *The Fundamental Concepts of Philosophy* and to a lesser extent *The Contributions to Philosophy (Of the Event)*, I set out Heidegger’s stratified account of boredom which, moving from a situative to an existential level of depth, involves increasingly pervasive levels of meaninglessness and monotony. Further, I discuss Heidegger’s early historical interpretation of boredom as the attunement that is definitive of the contemporary age, and his later concern that boredom in fact leads to alienation and indifference. Secondly, drawing on survey data collected by researchers in an ongoing project titled ‘Experiences of Social Distancing During the Covid-19 Pandemic: A Survey,’ I develop a novel phenomenological interpretation of the experience of pandemic boredom around the themes of meaninglessness and monotony. Thirdly, interpreting pandemic boredom from the unique vantage point of Heidegger’s philosophy of boredom, I argue that we should understand pandemic boredom as a situative attunement that is concerned with relative meaninglessness, which ultimately maintains our captivation with the everyday world. At the same time, I suggest, pandemic boredom can be seen to complicate Heidegger’s ontological distinction between situative and existential attunements in important ways and, in so doing, makes it difficult to determine the relative significance of different experiences of boredom; particularly when we consider the heterogeneous social, cultural and political conditions within which they unfold.

## The philosophy of boredom

Though many philosophers have reflected on the significance of boredom, Martin Heidegger’s 1929–1930 lecture course on *The Fundamental Concepts of Metaphysics* (Heidegger, 1995) stands as one of most influential and systematic philosophical accounts of the phenomenology of boredom; complemented (and to some extent complicated) by his discussion of indifference in his 1936–1938 notes *The Contributions to Philosophy (Of the Event)* (Heidegger, 2012). Two prominent interpretations that have critiqued and extended Heidegger’s philosophy of boredom include Lars Svendsen’s *A Philosophy of Boredom* (Svendsen, 2005) and a series of articles and books by Andreas Elpidorou including some co-authored with Lauren Freeman (Elpidorou, 2014, 2018a, b, 2020, 2021a; Elpidorou & Freeman, 2015, 2019). As is well known, Heidegger gives a stratified typology that differentiates between the first, second and third levels of boredom (*Langeweile*).^8^ At the first level, one is left in limbo and left empty *by* something determinate within the world, such as a train that has not yet arrived at the station. At the second level, one is bored *with* an indeterminate situation within the world such as a dinner party, which refuses itself in a way that is more diffuse and yet more pervasive. Following Svendsen’s interpretation in *A Philosophy of Boredom*, we can see that the first two levels of Heidegger’s typology constitute *situative* boredom, an emotion directed toward a particular object or situation within the world that has lost its meaning (Svendsen, 2005, pp. 110–111). Directed towards the world, situative boredom involves an experience of ‘relative meaninglessness,’ the refusal of something determinate or indeterminate to show up as significant, purposeful or consequential. At the third, existential level, however, profound boredom involves the world in its entirety becoming *boring for one*. Here the world as a whole ‘refuses itself’ in its meaninglessness, and one is thereby abandoned to oneself (Heidegger, 1995, pp. § 19–§ 38). As Svendsen emphasises, the third level of profound boredom constitutes *existential* boredom, a directionless, objectless mood, which involves the loss of meaning of the world as a whole (Svendsen, 2005, pp. 110–111). In this experience of ‘absolute meaninglessness’: “The meaning of human life collapses. The relationship of *Dasein* to the world disappears, and what remains is a nothing, all-encompassing lack…Boredom is dehumanizing by depriving human life of the meaning that constitutes it as a life” (Svendsen, 2005, p. 128).

Inherent in the German *Langeweile* (‘long while’), the three levels of boredom further involve an increasingly pervasive disturbance of temporal experience. At the first situative level of boredom, time slows down and stagnates, in a dragging that is experienced as paralysing. At the second situative level, time stands still; as the future and the past withdraw, and one finds oneself limited to the present, a static ‘now’ that merely persists, enduring without flowing. At the most profound, existential level of boredom, this present lengthens and extends in to an overwhelming and oppressive expanse. Here one finds that the entire horizon of time has withdrawn and refused itself. One finds oneself pushed to the limit or extremity of time, to the extent that they may feel “timeless” and “removed from the flow of time” altogether (Heidegger, 1995, p. 141/ GA 129/130, 213). Held out into atemporality, Svendsen writes that “one is caught in a vortex of immanence, where *Dasein* is no longer genuinely ec-static, i.e. transcending.” In this way, “Boredom is reminiscent of eternity, where there is no transcendence. Time collapses, implodes, into a vast, empty present” (Svendsen, 2005, p. 127).

Understood as such, Heidegger’s stratified analysis of situative and existential boredom is informed by his overall philosophical interpretation of affect in important ways. Firstly, throughout his interpretation of different affective phenomena, Heidegger consistently differentiates between attunements that turn one towards the world in which one is situated (for example fear, the first two levels of boredom, amazement and marvelling), and fundamental attunements (*Grundstimmungen*) that turn one away from the world and towards the question of Being and time (for example, joy, anxiety, profound boredom, holy mourning, shock, awe, and restraint, wonder, startled dismay, and releasement). Whilst Heidegger’s early discussions of inauthenticity and authenticity give the impression that this is a normative or moral distinction, it is intended first and foremost as an ontological one; concerned not with whether different attunements are good or bad, positive or negative, but whether they force a reckoning with the question of Being and time and thus whether or not they are *philosophically revelatory*.^9^ Accordingly, situative attunements such as the first two levels of boredom are derived from and grounded in existential attunements such as profound boredom, in the sense that being left in limbo and being left empty is profound boredom that has become entangled with the world. Thus whilst situative boredom is not of particular philosophical concern for Heidegger, it nevertheless has traces of the existential boredom upon which it depends.

Secondly, in both situative and existential boredom there is an important convergence of the experiences of meaninglessness and monotony. This correlation between attunements and temporality is not incidental for Heidegger but is again integral to his underlying philosophical interpretation of affect. On the one hand, Heidegger argues that the significance of attunements — how they enable the world to matter to us, to show up as threatening or captivating, familiar or unfamiliar, disturbing or comforting, meaningful or *meaningless* — must be interpreted on the basis of temporality. This is because temporality, the unified, three-fold structure of past, present and future, is what structures one’s existence and therefore makes it possible for one to find oneself disposed through attunements in the first place (Heidegger, 1962, pp. 390–391/ GA SZ, 340–341; p. 141/ GA 129/130, 213). On the other hand, Heidegger is also clear that the *way in which* temporal experience unfolds is in turn determined (*bestimmt*) by the different attunements (*Stimmungen*) with which one finds oneself affected. For Heidegger, “[a]ttunements temporalize themselves” by modifying the way in which the temporal structure of existence as a whole unfolds (Heidegger, 1962, p. 390/ GA SZ, 340). Depending on the particular attunement with which one is affected, therefore, temporal experience is subject to a “peculiar transformation” (Heidegger, 1995, p. 125/ GA 129/130, 189); past, present and future are denied and withheld in different configurations such that, whilst some dimensions become blocked, others are intensified. These modifications to the contours of temporal experience then have significant implications for the way in which one finds oneself in the world in any given attunement (see Hughes 2020a, b, 2022). It is in the sense of this inter-reliant ontological relation between attunements and temporality that the experiences of meaninglessness and monotony in boredom are necessarily intertwined for Heidegger. The denial of the past, the withholding of the future and the intensification of the present are what make it possible for boredom to disclose the world as meaningless. At the same time, boredom itself is what contorts and constricts temporal experience into the monotony of the immanent present.

The inherent unity of attunements and temporality is of further importance to Heidegger’s overall interpretation of affect because it enables him to put forward the idea that attunements are not a-historical, but historically referential, and grounded in the way different historical epochs unfold over time. Indeed, whilst the ancient epoch is defined by wonder, and the modern by doubt and confidence, Heidegger speculates in *The Fundamental Concepts of Metaphysics* (1929–1930) that boredom (along with doubt, despair, fear and hope) is one of the paradigmatic attunements of the contemporary epoch. He reflects:

Why do we find no meaning for ourselves any more, i.e., no essential possibility of being? Is it because an *indifference* yawns at us out of all things, an indifference whose grounds we do not know? Yet who can speak in such a way when world trade, technology, and the economy seize hold of a man and keep him moving?...What is happening here?, we ask anew. Must we first make ourselves interesting to ourselves again? Why *must* we do this? Perhaps because we ourselves have become *bored* with ourselves? Is man himself now supposed to have become bored with himself? Why so? *Do things ultimately stand in such a way with us that a profound boredom draws back and forth like a silent fog in the abysses of Dasein?* (Heidegger, 1995, p. 77/ GA 29/30, 115).

For individual Dasein, this indifference of all things and the refusal of the world as a whole is what compels one toward the abyssal ground of our existence— the question of nothing and thereby Being. Yet, as the attunement that also grounds the contemporary epoch, the experience of absolute meaninglessness and the confrontation with the meaning of existence in profound boredom has the potential to be philosophically revelatory of the significance of our contemporary moment.

And yet, by the 1936–1938 work *Contributions to Philosophy (Of the Event)*, Heidegger is more hesitant. In this text he expresses concern that, the ‘calculation,’ ‘speed’ and ‘massiveness’ of homogenized and technologized societies may in fact overwhelm any revelatory capacity that boredom has and lead instead to a pervasive sense of indifference, disillusionment and self-alienation (Heidegger, 2012, pp. 95–98/ GA 65, 119–124). In this sense boredom appears to no longer be an existential, fundamental attunement for Heidegger, but a necessarily *situative* one, involving an experience of relative meaninglessness that merely maintains our captivation with the everyday world. Heidegger’s ambivalence is reflective of the divisiveness of this question in the philosophy of boredom more generally,^10^ and, as we will see, it has important implications for the way in which we are to interpret and understand the philosophical significance of pandemic boredom.

## A phenomenology of pandemic boredom

As the magnitude and severity of the Covid-19 pandemic has become increasingly apparent, researchers in many different disciplines have sought to document the impact of both the virus and the government-led interventions put in place to mitigate against it. In setting out an account of pandemic boredom I will draw from one particularly informative example, an ongoing, multi-disciplinary research project titled: ‘Experiences of Social Distancing During the Covid-19 Pandemic: A Survey.’ Led by Tom Froese at the Okinawa Institute of Science and Technology Graduate University in Japan, the project involves researchers in psychology, philosophy, psychiatry, medicine and anthropology from the University of Bristol, the University of Birmingham, and the University of York, and is interested in understanding first-person experiences of the pandemic through the lenses of both cognitive science and phenomenology. From June to July of 2020, the team collected survey responses from some 2543 participants in Japan, Mexico and the UK, asking them a wide-range of questions about their experiences of social distancing measures during the early stages of the pandemic which, at the time of writing is still ongoing. These data have been made publicly available and will inform the basis of my account.^11^

As the researchers note in their summary report, boredom is a prominent theme in the survey responses, and is often experienced in connection with a loss of temporal flow (Froese et al., 2021, p. 5). In referring to ‘boredom,’ ‘boring,’ or ‘bore,’ respondents describe pandemic boredom as involving feelings of confusion (ES_00_0650); strangeness (ES_MX_1558); impatience, despair (ES_MX_0600); anxiety and desperation (ES_00_0733). They describe frustration at being idle (ES_MX_0600) and having wasted time (ES_00_0733); at feeling trapped and wondering whether it will ever end (ES_MX_1678). They report having difficulty thinking or concentrating (ES_MX_1678); and sleeping and eating either too much or too little as a result of being bored (EN_UK_1105, EN_UK_1252, EN_UK_1298). Framed by Heidegger’s phenomenological interpretation of boredom, these descriptions of emptiness, restlessness, frustration, weariness and indifference in pandemic boredom can be understood in terms of the themes — both found in Heidegger’s account — of meaninglessness and monotony.

These inter-related ideas of diminished meaning and monotonous time become particularly apparent when focusing upon responses to Question 57: ‘Have you noticed changes in your experience of time?’ With regards to the loss of meaning, respondents repeatedly emphasise the lack of meaningful events to anchor things that have happened in the past (EN_UK_1081), to mark the passage of time in the present (EN_UK_1691), or to look forward to in the future (EN_UK_1081). When there are no signposted events “like a holiday or trips away or visits to a restaurant or cinema, the days and weeks seem to be merging into one another” (EN_UK_0432). As this respondent notes: “This is like a prison sentence. No life goals means life is very boring and very slow” (EN_UK_1231). Related to the experience of meaninglessness that arises from the lack of events is the sense that, without projects or possibilities to work towards, one feels that one has not accomplished anything, despite having long, empty days with seemingly lots of time in which to do so. This is a source of significant frustration for many respondents: “Sitting in front of my laptop all day trying to work, I am always wondering where the time went at the end of the day. I feel that I lost a day as I wasn’t engaged in any activities that were meaningful to me” (EN_UK_1083); “I don’t feel like I’m achieving very much” (EN_UK_0145); “I got very little done in this time” (EN_UK_2392).

This loss of meaning is intricately related to the experience of temporal disruption. In describing changes to the flow of lived time, a majority of respondents depict time as both speeding up and slowing down. Whilst the days can stretch out endlessly, the weeks and months seem to elapse very quickly. As this respondent writes:

Yes. I feel like the year has been standing still and am constantly amazed at the months passing by. It feels like time has stretched so that the days seem longer, but also contracted so that both the weeks and the weekends seem to go by very quickly. It’s a strange experience of time! (EN_00_1434).

Both fast and slow, some respondents describe the form or structure of time as losing its shape, as past, present and future appear to merge, blur, blend or drift: “Yes. In the last month time seems [to be] running incessantly, as if there is no distinction between morning and night, today and yesterday” (EN_00_0155). This disturbance of the structure of time can be disorienting. “Every day seems to blend into one” (EN_UK_0060) and one begins to lose track of time. As this respondent notes: “I have much less sense for the passage of time, and have a hard time doing things like estimate how long has passed since an event (two weeks? A month?). I say often that ‘time is meaningless now’” (EN_00_1440). As a result of this temporal disorientation, linear, chronological time becomes increasingly irrelevant and arbitrary. For instance, a number of respondents note that they no longer wear a watch, and that the only thing that marks the time is the allocated time-slot for online shopping deliveries or the weekly bin night: “I literally have no idea what day it is until bin day comes around. I go to bed when I’m ready and wake up when I feel. Time doesn’t really matter much” (EN_UK_0086). Together, these different aspects of temporal disturbance constitute the monotony and repetitiveness that is definitive of the restricted experience of time. In describing this recurrent sameness, several respondents make reference to Ramis’ film *Groundhog Day* or Beckett’s *Waiting for Godot*: “It feels as if I am permanently in a waiting room but I don’t know what I am waiting for — Godot perhaps!” (EN_UK_0413).

Marc Wittman gives a helpful explanation of this interplay of meaninglessness and monotony in pandemic boredom in his recent article ‘Subjective Passage of Time during the Pandemic: Routine, Boredom, and Memory’ (Wittmann, 2020). Drawing on the cognitive approach to time perception (Block & Zakay, 1997; Wearden, 2016), Wittman argues that pandemic boredom (like depression) induces an experience analogous to Pöppel’s time paradox (Pöppel, 1988, p. 88), wherein *prospective time perception* (which is a judgement of *duration*) and *retrospective time perception* (which is a judgement of a time *interval* that has already elapsed) appear to contradict one another. Specifically, the repetitiveness of social restrictions mean that in judgements of duration, time seems to pass very slowly. Yet, because nothing has happened and there are no meaningful projects or events with which to structure the passage of time, the time interval that has elapsed appears to have passed very quickly when reconstructed from memory, and a ‘quarantine paradox’ emerges as a result.^12^

## The significance of pandemic boredom

Heidegger’s phenomenological interpretation of boredom and the defining themes of meaninglessness and monotony provide an important framework through which to cohere the descriptions of emptiness, restlessness, frustration, weariness and indifference in pandemic boredom. The question then arises as to how Heidegger’s account might help us to understand its significance: as a situative confrontation with relative meaninglessness that upholds our absorption in the everyday world, or as an existential confrontation with absolute meaninglessness that forces us to take up the question of our existence. On the face of it, the experiences of pandemic boredom described in the survey data are essentially *situative*; involving a confrontation with relative meaninglessness and monotony that I suggest can be seen to correspond to the first and second levels of boredom in Heidegger’s stratified typology. In particular, whilst the suffering can be overwhelming, intense and prolonged in pandemic boredom, that in the face of which the respondents are bored is necessarily attached to something within the world, namely: the *absence* of significant, purposeful or consequential intersubjective projects and possibilities, both determinate and indeterminate, that are together constitutive of one’s familiar world. Given that pandemic boredom does not appear to involve the confrontation with absolute meaninglessness that is required if one is to take up the question of existence, it is difficult to see on a Heideggerian interpretation how it could be considered philosophically revelatory. On the contrary, pandemic boredom arguably realizes Heidegger’s ambivalent concerns, expressed in the *Contributions to Philosophy (Of the Event)*, that in homogenized and technologized societies boredom merely reflects and then intensifies our distracted and restless immersion in the world. Indeed, it isn’t hard to see how Heidegger could view both the pandemic and the attempts to mitigate against it as being a product of a ‘levelled down’ modern society; where calculation, speed and massiveness overwhelm any revelatory capacity that boredom may have had, leading instead to the pervasive sense of indifference that is evident in many survey responses.

Beyond this straightforward explanation, however, it is my view pandemic boredom can be seen to expose and then exceed the distinctive methodological limitations of Heidegger’s phenomenological interpretation of boredom in several ways: with regards to the ontological distinction between situative and existential attunements and then in terms of the relative significance of different experiences of boredom. Firstly, pandemic boredom problematizes Heidegger’s ontological distinction between situative and existential attunements, which hinges on whether attunements turn one *toward* or *away from* the everyday world. Whilst pandemic boredom *is* situative in that it concerns the absence of significant intersubjective projects and possibilities that constitute one’s familiar world, this categorization is complicated by the fact that the pandemic has profoundly destabilized the tacit structure of the everyday world in a more general sense. Schools, universities, shops, restaurants, playgrounds, cinemas and churches have been closed; travel has been prohibited; and significant occasions such as weddings, funerals and birthday parties have been postponed. In this way, it is not only aspects of *one’s* familiar world that have been withheld, but substantial aspects of the familiar world *itself* that have been indefinitely suspended; withdrawn from many, if not all, to varying extents.^13^ Whilst pandemic boredom is still situative, therefore, the stable points of reference that ordinarily demarcate the ‘limit’ between the familiar and unfamiliar world can no longer be presupposed, which leads to significant ambiguity. As a result, pandemic boredom is not constrained by a relative attachment to the everyday world; it is wider-ranging and more diffuse, making it harder to distinguish from existential boredom, despite the fact that it is still very much oriented towards the ‘world.’ If there is no intact everyday world for one to be either absorbed by or estranged from, it becomes difficult to sustain the Heideggerian distinction between situative and existential boredom, which then weakens the assumption that pandemic boredom cannot be philosophically revelatory. At stake here is the implication that, if there is no unified world from which one can find oneself displaced, no ‘outside’ from which the world as a whole might be lit up, then there is no unique or privileged position from which to grasp it philosophically for Heidegger.

Secondly, and relatedly, the instability of the world and the complication of the distinction between situative and existential attunements then makes it difficult to discriminate between the relative significance of different experiences of pandemic boredom; particularly when we consider the heterogeneous social, cultural and political conditions within which they unfold.^14^ Drawn from examples of being bored at the train station and a dinner party, Heidegger’s interpretation of boredom struggles to account for how different people’s concrete factical situations, which have ensured the unequal distribution of the pandemic’s adverse impacts upon mental health, relationships, work, education, and finances more generally, might influence how we interpret the significance of different people’s experiences of pandemic boredom (Bambra et al., 2021; Fineberg et al., 2021; Gadermann et al., 2021; Niedzwiedz et al., 2021; Reme et al., 2022). Considering one particularly salient example, many survey respondents emphasise how the quietness and stillness brought about by social distancing measures has led to a heightened appreciation of meaningful relationships with partners, children, family and friends, and has reinforced the importance of not taking these for granted: “We seem to be getting along better, if anything. We usually hare around, between work and kids activities, we have little time for family life and relaxation. We’ve done lots of nothing — it’s nice and once things lift we’ll try to keep a slightly slower pace” (EN_UK_0046). A significant number of respondents also describe the pandemic as giving them more clarity around those relationships that are important to them and those that aren’t: “Lockdown has been one of the most creative and socially intense times of my life. It has given me the space to think about how I want to interact with others, try it out, and end up with ways that I think are best” (EN_UK_1889). Other respondents report an increased feeling of connection with neighbours and trust of their community due to a sense that everyone is in the same situation (EN_UK_0054) and that people are looking out for each other (EN_UK_0445). At the same time, social distancing measures have exposed the fraughtness of interpersonal relationships with many respondents describing an increase in tension within their households: “Yes, very much. More arguments and destructive remarks, less warmth and love in household as our natural balance of relationships is so deprived” (EN_UK_1074). For some respondents, tension has also increased within their wider circle of family and friends (EN_UK_0495). Many also report a more pervasive feeling of disconnection with neighbours and a greater distrust and wariness of their community: “The pandemic has absolutely reinforced my distrust of other people, previously selfish and self-centred and multiplied now” (EN_UK_0772). In this sense, whilst for some the absence of the familiar world has renewed the possibility of meaningful interpersonal relationships, for others it has revealed their meaninglessness, fragility and contingency. Similarly contingent upon these differential situations, pandemic boredom has the potential to be both constructive and destructive, transformative for some in taking up responsibility for shaping their lives in a meaningful way, and stifling for others. Yet, with the ontological difference between situative and existential attunements in question, it becomes difficult to discriminate between the relative significance of these different experiences of pandemic boredom. Beyond their intrinsic importance, that is, in describing the way in which different people in different circumstances find themselves affected, can some situative attunements be philosophically revelatory and not others? How do we discriminate?^15^ Deprived of the privileged philosophical vantage point that existential attunements supposedly afford, Heidegger’s interpretation of boredom is forced back onto psychological explanations of the relative significance of different experiences of boredom, and thus must answer to the ongoing and unresolved attempts at distinguishing between state and trait, common and modern, exogeneous and endogenous, or non-pathological and pathological boredom.^16^

To conclude, Heidegger’s phenomenological interpretation of boredom provides an important framework through which to understand the experiences of pandemic boredom according to the defining themes of meaninglessness and monotony. However, there are important ways in which pandemic boredom exposes then exceeds the methodological limitations of Heidegger’s interpretation of boredom’s significance, particularly through the problematization of the ontological distinction between situative and existential attunements and then the relative significance of different experiences of boredom. The most significant question pandemic boredom raises for Heidegger’s interpretation of boredom is that: if there is no philosophically privileged vantage point through which to grasp the world as a whole, then how can we determine the relative significance of different experiences of boredom, beyond their importance in describing the way in which we find ourselves in the world? In this way, the phenomenological study of pandemic boredom demonstrates the significant value of Heidegger’s philosophical interpretation of boredom, as well as the need to move towards a more critical appropriation of his work.

## Footnotes

1 These two aspects of boredom — the loss of meaning and the slowing down of time — are represented by the etymologies of the English boredom and the German *Langeweile* respectively. Found in the the Middle-English ‘bore,’ boredom is not derived from the German *Langeweile* but from the Old High German boron and the German bohren, meaning ‘to cut with a sharp point, pierce, bore,’ and capturing the idea of absence or emptiness. Correlatively, the German *Langeweile* — literally ‘long while’ — denotes the slowing down of time that is inherent to the experience of boredom.

2 For a multi-disciplinary overview of boredom studies see for example (Haladyn & Gardiner, 2017a; Spacks, 1995; Toohey, 2011). In addition to the International Society of Boredom Studies a number of other research groups dedicated to the study of boredom have recently formed, including the Boredom Lab at York University and The Danckert Lab ‘Dissecting Boredom’ project at the University of Waterloo. See (Danckert & Eastwood, 2020).

3 See (Arslan & Yıldırım, 2021; de Jong et al., 2020; Milman et al., 2020; Salicru, 2021; Trzebiński et al., 2020).

4 See (Droit-Volet et al., 2020; Grondin et al., 2020; Grove et al., 2022; Holman & Grisham, 2020; Irons, 2020; Linker, 2020; Ogden, 2020; Pardes, 2020; Schnalzer, 2020; van der Werff, 2020; Virna & Brahina, 2020; Wessels et al., 2022; Wittmann, 2020).

5 I will use the term ‘pandemic boredom’ to refer to the experiences of boredom that have occurred as a result of, or during, the Covid-19 pandemic.

6 Within the expanding field of boredom studies, psychologists in particular have emphasised the distinctive importance of pandemic boredom as giving insight into the distinction between trait and state boredom, the role of emotional regulation and well-being, and whether or not there is a correlation between boredom proneness and adherence to social distancing measures. See for example (Bieleke et al., 2021; Boylan et al., 2021; Martarelli et al., 2021; Wolff et al., 2020) as well as the recent Special Issue of *Behavioural Sciences* on ‘Boredom in the Covid-19 Pandemic’ edited by James Danckert (2022).

7 See Heidegger’s discussion of *Das Man* (‘the They’) in *Being and Time* for further clarification of the idea of being absorbed in the everyday world (Heidegger, 1962/ GA SZ).

8 Whilst there are some important exceptions (O’Brien, 2014), the majority of boredom researchers emphasise its heterogeneous or stratified nature; including Wendell O’Brien in this helpful development of his earlier interpretation (O’Brien, 2021). As Chruszczewski (2020) and Svendsen (2005) demonstrate in their detailed taxonomies, there are many different typologies of boredom, many of which indicate variation in depth, duration and severity. For example, beyond the well-known distinction between state boredom and trait boredom in psychology (Todman, 2003), sociologist Martin Doehlmann differentiates situative boredom, the boredom of satiety, existential boredom, and creative boredom (Doehlemann, 1991, pp. 22–23); writer Gustave Flaubert (1976), common boredom (*ennui commun*) from modern boredom (*ennui modern*); philosopher Jerome Neu (1998), exogenous or reactive boredom from endogenous boredom; and psychoanalyst Otto Fenichel (1951), non-pathological boredom from pathological boredom. As Chruszczewski notes, there are important correlations between situative, common, exogenous and non-pathological boredom on the one hand and existential, modern, endogenous and pathological boredom on the other (Chruszczewski, 2020, pp. 239–240). In a recent article, Elpidorou sets out some of the problems posed by the heterogeneity of boredom and proposes a functional solution (Elpidorou, 2021b).

9 Whilst Elpidorou’s early work on boredom engages Heidegger’s existential-ontological interpretation of affect, his own interpretation has moved increasingly towards the normative, moral and psychological implications of boredom which Heidegger himself eschews. This fundamental difference is evident in his recent book, *The Moral Psychology of Boredom*, wherein he describes profound boredom as a heterogenous phenomenon that can vary according to its object, scope, frequency, duration, intensity and cause (Elpidorou, 2021c, pp. 5–6). This is in sharp contrast to Heidegger, who delineates profound boredom on the ontological grounds that it turns one away from the world and towards the question of Being and time in a way that is philosophically revelatory.

10 For helpful overviews of the historical development of the philosophy of boredom see (Haladyn & Gardiner, 2017b; Svendsen, 2005).

11 My analysis of pandemic boredom draws on the initial data set which was collected relatively early on in the pandemic. From April to July 2021 the team re-interviewed a large proportion of the participants using the same questions, the responses from which have recently been made publicly available (James et al., 2022). Whilst it is beyond the scope of this article, it would be useful to compare and contrast the experiences of pandemic boredom across these two data sets.

12 Interestingly, the time paradox need not always be a negative experience. See for example Claudia Hammond’s concept of the ‘Holiday Paradox’ in *Time Warped* (Hammond, 2012).

13 A related concern here is that Heidegger’s individualistic conception of boredom does not allow for a collective turning away from the familiar world. That is to say, being withdrawn from ‘the world’ depends on others continuing to be absorbed in it, which has not been possible in the pandemic.

14 For a related critique see (Golob, 2017, pp. 262–263; Okrent, 1999, p. 73).

15 Of course, one might be sceptical of the very idea that any affective phenomena are philosophically revelatory, however, to address that concern would be to go beyond Heidegger’s distinctive project and therefore the scope of this paper.

16 See note 6.
